# Localization of the quantitative trait loci related to lodging resistance in spring bread wheat (Triticum aestivum L.)

**DOI:** 10.18699/VJGB-22-82

**Published:** 2022-11

**Authors:** I.N. Leonova, E.V. Ageeva

**Affiliations:** Institute of Cytology and Genetics of the Siberian Branch of the Russian Academy of Sciences, Novosibirsk, Russia; Siberian Research Institute of Plant Production and Breeding – Branch of the Institute of Cytology and Genetics of the Siberian Branch of the Russian Academy of Sciences, Novosibirsk, Russia

**Keywords:** spring wheat, lodging, plant height, upper internode diameter, GWAS, QTL, яровая пшеница, полегание, высота растения, диаметр верхнего междоузлия, GWAS, QTL

## Abstract

The yield and grain quality of spring and winter wheat signif icantly depends on varieties’ resistance to lodging, the genetic basis of this trait being quantitative and controlled by a large number of loci. Therefore, the study of the genetic architecture of the trait becomes necessary for the creation and improvement of modern wheat varieties. Here we present the results of localization of the genomic regions associated with resistance to lodging, plant height, and upper internode diameter in Russian bread wheat varieties. Phenotypic screening of 97 spring varieties and breeding lines was carried out in the f ield conditions of the West Siberian region during 2017–2019. It was found that 54 % of the varieties could be characterized as medium and highly resistant to lodging. At the same time, it was noted that the trait varied over the years. Twelve varieties showed a low level of resistance in all years of evaluation. Plant height-based grouping of the varieties showed that 19 samples belonged to semi-dwarfs (60–84 cm), and the rest were included in the group of standard-height plants (85–100 cm). Quantitative trait loci (QTL) mapping was performed by means of genome-wide association study (GWAS) using 9285 SNP markers. For lodging resistance, plant height, and upper internode diameter, 26 signif icant associations (–log p > 3) were found in chromosomes 1B, 2A, 3A, 3D, 4A, 5A, 5B, 5D, 6A, and 7B. The results obtained suggest that the regions of 700–711 and 597–618 Mb in chromosomes 3A and 6A, respectively, may contain clusters of genes that affect lodging resistance and plant height. No chromosome regions colocalized with the QTLs as sociated with lodging resistance or upper internode diameter were found. The present GWAS results may be important for the development of approaches for creating lodging-resistant varieties through marker-assisted and genomic selection.

## Abstract (ru)

Урожайность и качество зерна яровой и озимой пшеницы в значительной степени зависят от устойчивости сортов к полеганию. Генетический контроль устойчивости к полеганию носит количественный характер и контролируется большим числом генных локусов, поэтому изучение генетической архитектуры данного признака необходимо для создания и усовершенствования современных сортов. В работе представлены результаты по выявлению геномных районов, ассоциированных с устойчивостью к полеганию и сопряженными с ним признаками «высота растения» и «диаметр верхнего междоузлия» у российских сортов яровой мягкой пшеницы. Фенотипический скрининг 97 яровых сортов и селекционных линий мягкой пшеницы был проведен в полевых условиях Западно-Сибирского региона в 2017–2019 гг. Установлено, что 54 % сортообразцов можно охарактеризовать как средне- и высокоустойчивые к полеганию, при этом отмечено варьирование признака по годам. Двенадцать сортов проявляли низкий уровень устойчивости во все годы проведения испытаний. Группировка растений по высоте показала, что 19 образцов относятся к полукарликам (60–84 см), остальные вошли в группу низкорослых растений (85–100 см). Картирование локусов было проведено с помощью полногеномного ассоциативного (GWA) анализа с использованием 9285 маркеров SNP. Для признаков «устойчивость к полеганию», «высота растения» и «диаметр верхнего междоузлия» найдено 26 значимых ассоциаций (–log p > 3) в хромосомах 1B, 2A, 3A, 3D, 4A, 5A, 5B, 5D, 6A и 7B. Полученные результаты позволяют предположить, что районы 700–711 и 597–618 Mb хромосом 3А и 6А соответственно могут содержать кластеры генов, влияющих на устойчивость к полеганию и высоту растения. Не обнаружено районов хромосом с колокализацией локусов, ассоциированных с устойчивостью к полеганию и диаметром верхнего междоузлия. Данные GWA анализа могут иметь значение для разработки методов создания устойчивых к полеганию сортов с помощью маркер-ориентированной и геномной селекции.

## Introduction

Spring bread wheat (Triticum aestivum L.) is one of the main food crops grown in Western Siberia taking about 40 % of all agricultural lands (5.5 mln ha). According to the Russian Statistics Agency, the wheat yield has been growing recently in the region, comprising in different years from 21 to 28 cwt/ha ( Agriculture in Russia. https://rosstat.gov.ru/folder/210/document/13226. Lodging is one of the important factors resulting in a serious yield loss and reducing the technological quality of the grain. In lean years, early lodging in spring soft wheat can lead to a yield loss of up to 20–50 % in the milky ripeness phase, and up to 15 % – in the wax ripeness phase (Stapper, Fischer, 1990; Zhuchenko, 2004; Khobra et al., 2019). Lodging com plicates mechanized harvesting, which results in additional yield loss. The weather conditions monitoring carried out in Western Siberia from 1976 to 2016 has shown that the regional climate has become more extreme, so its increased frequency of gales, showers and thunderstorms significantly reduces the yield (Kharyutkina et al., 2019). Considering this unfavorable weather conditions, it becomes essential to create the wheat varieties that are resistant to lodging

Lodging resistance is a trait that depends on a number of features, the most important of them being the stem’s ana tomical and morphological properties. So far, it has been found that the plant height is crucially important for the trait in question. Discovering the genes of reduced height (Rht) as well as introduction of the most effective genes and their alleles (Rht-B1b, Rht-D1b, Rht8) into the wheat genome have resulted in creation of the varieties resistant to lodging (Khobra et al., 2019; Liu et al., 2022). Meanwhile, a number of stu dies have demonstrated that reducing the plant’s height below a certain value leads to reduced grain size, 1000-grain weight and a worse yield in general (Miralles, Slafer, 1995; Flintham et al., 1997; Li et al., 2006). In unfavorable weather conditions, the alleles of the Rht-B1b and Rht-D1b can have a negative effect on the plant’s coleoptile length and root size preventing proper rooting and reducing drought resistance (Rebetzke et al., 1999; Ellis et al., 2004; Yan, Zhang, 2017). The undesirable effects of the Rht gene alleles also include reduced nitrogen content in grain and a longer heading time, resulting in worse yield and grain quality (Casebow et al., 2016; Sukhikh et al., 2021). Apart from the stem’s height, its other parameters are of crucial importance, e. g., it has been found that the culm’s diameter, wall thickness and weight, number of vascular bundles and mechanical tissue sizes may determine wheat resistance to lodging (Berry et al., 2003; Zakharov et al., 2014).

Being of quantitative character, lodging is controlled by a large number of genes that complicates the creation and se lection of resistant genetic lines using the methods of classical breeding and phenotyping. Many researchers believe pheno typic assessment of lodging resistance may be controversial since lodging occurs at different stages of plant development and its degree is affected by certain external factors (Atkins, 1938; Hai et al., 2005). On the other hand, marker-based analysis and identification of the genome regions associated with lodging may be used for indirect selection of the varieties unsusceptible to lodging.

The modern technologies for mapping of genes and quan titative trait loci (QTL) enable one to determine the chromo somal and genomic localization of target loci and the archi tecture of their quantitative traits. For the time being, genome- wide associated studies (GWAS) have become one of the most commonly applied approaches for mapping the QTLs of agronomically important traits. The effectiveness of the technique has been confirmed to detect and localize the loci responsible for wheat resistance to biotic (Aoun et al., 2021; Kokhmetova et al., 2021) and abiotic (Wang N. et al., 2019; Pshenichnikova et al., 2021) stress factors and their effect on the yield capacity (Luján Basile et al., 2019; Gahlaut et al., 2021), grain protein content, and baking quality (Battenfield et al., 2018; Leonova et al., 2022).

Currently, there have been just a few studies using GWAS for mapping the loci correlating with lodging resistance or responsible for related stem characteristics in the wheat (Ce ricola et al., 2017; Malik et al., 2019; Akram et al., 2021), so the objective of the present study was (1) to perform com parative screening of spring soft wheat varieties for lodging resistance, plant height, and upper internode diameter; (2) to detect the potential genome regions associated with lodging or its related stem characteristics using the association map ping technique.

## Materials and methods

Plant material and phenotyping. A collection of 97 varieties and breeding lines of spring soft wheat (T. aestivum L.) from different breeding centers of the Russian Federation that have been recommended for cultivation in Western Siberia was used in this study. Detailed information on the plant material can be found in Suppl. Material 12

Supplementary Materials are available in the online version of the paper: http://vavilov.elpub.ru/jour/manager/files/Suppl_Leonova_27_7.pd


The plant material was grown in the field of Siberian Research Institute of Plant Production and Breeding, a Branch of the Institute of Cytology and Genetics of the Siberian Branch of the Russian Academy of Sciences (Novosibirsk Region, 54.9191° N, 82.9903° E) for three seasons (2017–2019). The samples were sown manually following the systematic method in two replications in plots of 1 m wide, 60 seeds in a row, and 25 cm between rows. The plants’ lodging was estimated during the wax ripeness phase according to the grading scale (Shamanin, Truschenko, 2006): 1 = very strong lodging, mechanized harvesting impossible; 2 = strong lodging; 3 = medium lodging, the stems are at 45° to the soil surface; 4 = weak lodging, the stems are barely inclined; 5 = no lodging. Height-based grouping was carried out as indicated in the methodological recommendations of the N.I. Vavilov All-Russian Institute of Plant Genetic Resources (VIR) (Guidelines for Studying…, 1987). To measure the upper internode diameter, stem crosssections were fixed in ethanol (96 %) and dyed in 1 % safranin solution (Safranin O, LLC ‘Dia-M’) to be photographed using a stereoscopic microscope Altami СМ0655 (LLC ‘Altami’) equipped with a camera Altami UCOS5100KPA. For statistical processing of the results, at least 10 plants of every sample were used.

The weather conditions within the years of investigation were, in general, favorable to yield formation (Suppl. Material 2). In the summer of 2017, 278 mm of precipitation was registered, in 2018 – 380.3 mm and in 2019 – 194.7 mm, the long-run annual average being 220 mm. According to the data of the Ogurtsovo agrometeorological station, the 2017 vegetation period was characterized by temperature fluctuations and often rains. In May and June of 2017, the temperature regime exceeded its long-run annual average, and there were not enough rains in the third decade of May and the second decade of June (10.5 mm, 65 % of the normal rate). The precipitation rate in July was 101 mm, the first decade being most rainy (49 mm). Selyaninov’s Hydrothermic Coefficient (HTC) comprised 0.9. The meteorological conditions in August remained within the normal rates, the third decade being characterized by insufficient precipitation (9.3 mm, 40 % of the normal rate). The average temperature during the summer months was 18.2 °С, which exceeded the long-run annual average by 0.6 °С.

The 2018 vegetation period was marked by lower temperatures in May (averagely, 5 °С below the normal range) and excessive precipitation in May–June if compared to the other seasons. During these two months, the precipitation rate comprised 211.4 mm being 80 % of the seasonal precipitation rate. May’s HTC was 10.2 and it reduced to 2.8 in June, whose temperature regime and precipitation level matched the long-run annual average. In August, precipitation deficiency was observed (–33.3 mm, HTC = 0.4).

The 2019 vegetation period was marked by unstable weather conditions due to uneven precipitation fallout, and temperature fluctuations in the second half of the period. The weather in May and July was rainy (HTC = 2.3 and 1.4, respectively). In June and August, a small drought was observed (HTC = 0.7 and 0.5, respectively).

DNA extraction, genotyping and GWAS. DNA was extracted from 5–7 day-old seedlings following a modified protocol as per Kiseleva et al. (2016). For the purposes of genotyping, the obtained DNA samples were purified in Bio- Silica microcolumns as per the manufacturer’s instructions. DNA concentrations were detected using a NanoDrop M2000 spectrometer (Thermo Scientific). Genotyping was carried out using a Triticum aestivum (wheat) genotyping Illumina Infinium 15K chip comprised of 13 006 SNP markers by the TraitGenetics company (Germany, www.traitgenetics.com).

The number of the polymorphic markers included in GWAS comprised 9235. Before the study, the markers were filtered and those with allele frequency of less than 5 % or not amplified in 20 % or more samples were excluded from the analysis, which was performed using a mixed linear model (MLM) in the TASSEL v. 5.2.70 software (Bradbury et al., 2007). The analysis considered population structure (Q-matrix) and genetic kinship (K-matrix), the first calculated using a Bayesian algorithm implemented in the STRUCTURE 2.3.4 software (Pritchard et al., 2000). The probable subscluster number was estimated using the Delta K (ΔK) statistics (Evanno et al., 2005) in the Structure Harvester web program (Earl, vonHoldt, 2012). The K-matrix was calculated using TASSEL v. 5.2.70. To find statistically reliable associations, the Benjamini– Hochberg method (1995) and FDR control at p <0.001 were applied. The chromosomal localization of the SNP markers was determined as per The IWGSC RefSeq v1.0 annotation (https://triticeaetoolbox.org) and the consensus maps of wheat chromosomes (Wang S. et al., 2014).

Statistical analysis of the obtained results was carried out in the STATISTICA v. 10 software (http://statsoft.ru/). To estimate the statistical reliability between the averaged values of two sampled populations, Student’s t-test was applied. The relation between lodging resistance, plant height and upper internode diameter was determined using Spearman’s correlation. The contributions of genotype and environment to trait manifestation were estimated using the ANOVA, whose statistical reliability was assessed through F-test. The heritability (H 2) was calculated based on the following formula:

**Formula 1.**
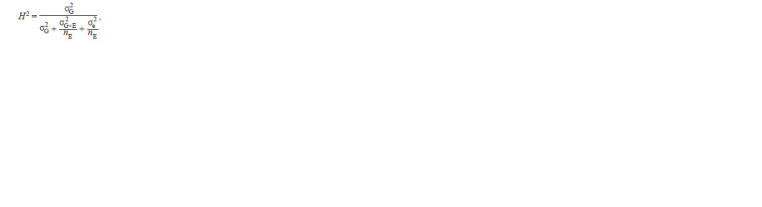
Formula where σ2 G, σ 2 G×E, σ2 e are the mean square deviations (SDs) of the genotype, genotype/environment interaction and residual variance, respectively, and nE is the number of vegetation seasons.

## Results

Phenotyping

**Fig. 1.**
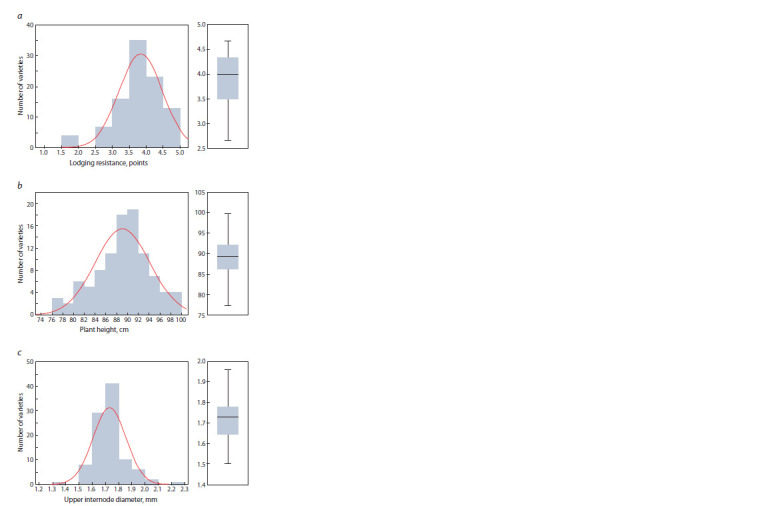
Histograms and boxplots to illustrate the distribution of spring soft wheat varieties relative to their (a) lodging resistance, points; (b) plant height, cm; (c) upper internode diameter, mm.

Estimating the varieties’ resistance to lodging within a 3-year period demonstrated that 53 out of 97 varieties could be related either to moderate or resistant kinds (>3.5 out of 5 points), and the trait varied from year to year (Table 1, Fig. 1, a). The highest degree of lodging was observed in 2018, which was related to the high precipitation level in the summer period, so the year did not produce a single variety with the highest level of resistance (5 out 5). Eight varieties (Novosibirskaya 29, Novosibirskaya 67, Novosibirskaya 91, Krasnoyarskaya 90, Vesnyanka 8, Mariinka, Salimovka, and Aleshina) demonstrated a high level of lodging resistance (4–5 points) in every year of the experiment. Unlike the above mentioned, twelve varieties (Saratovskaya 29, Saratovskaya 42, Lutescens 62, Altayskii prostor, Rosinka 2, Tulaikovskaya stepnaya, Lutescens 85, Surenta 6, Lutescens 840, Kinelskaya 40, Latona, and Volgouralskaya) had low lodging resistance (1–3 points) within the years of experiment.

**Table 1.**
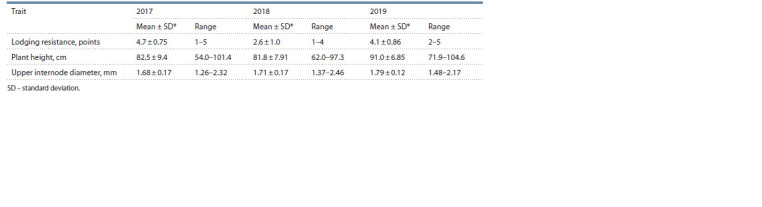
Characteristics of spring soft wheat varieties in relation to lodging resistance, plant height and upper internode diameter based on trait assessment in years 2017–2019

The plants’ height and upper internode diameter varied from 54 to 105 cm and from 1.26 to 2.46 mm, respectively, and depended on a vegetation season (see Table 1, Fig. 1, b, c). In the years 2018/19, the height varied less than in 2017, which means the characteristic depended on the soil and climate conditions. Grouping the plants by their height as per VIR Methodological Recommendations showed 19 varieties were semi-dwarfs (60–84 см) while the others comprised a group of dwarf plants (85–100 cm).

The ANOVA demonstrated that it was the genotype (G), environmental factors (E) and their interaction (G × E) that statistically contributed to the phenotypical manifestation of the said traits (Suppl. Material 3). The heritability was high for the plant height (78 %), while for the lodging resistance and upper internode diameter it comprised 51 and 59 %, respectively, which confirms the significant effect of the environmental factors on the phenotypical manifestation of the traits. Since none of the traits had normal distribution, their correlations were analyzed using Spearman’s rank correlations that showed statistically significant negative correlations between the lodging and height (r = –0.48***) and positive – between the lodging and upper internode diameter (r = 0.35***). The correlations between the height and the diameter were found to be weak (r = 0.20**).

GWAS

**Fig. 2.**
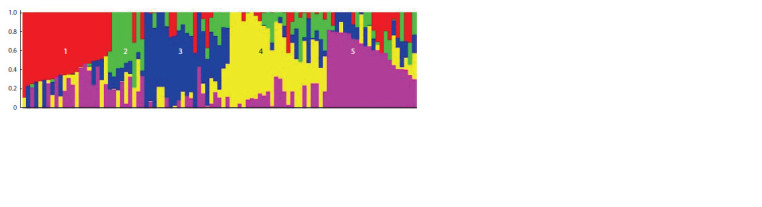
Population structure of the spring soft wheat varieties based on their SNP-marker genotyping results. The vertical axis marks the coefficients assigning a sample to a certain subcluster. Cluster compositions can be found in Suppl. Material 1.

The data analysis performed in the STRUCTURE software enabled us to subdivide the investigated varieties into five subclusters including 22, 7, 20, 25 and 23 genotypes, respectively (Fig. 2). It is noteworthy that this clustering did not match the plants’ origins as described by their originators (see Suppl. Material 1)

To detect genetic-marker associations with the considered characteristics, 9235 polymorphic SNP markers were used. The numbers of the markers mapped in the chromosomes of genomes A, B and D differed significantly, the smallest one registered for the chromosomes localized in homoeological group 4 (Suppl. Material 4). For 607 markers, data on their localization on the genetic and physical maps of wheat chromosomes were absent. The GWAS based on the estimation results of three vegetation seasons found 26 SNP markers that were significantly ( р < 0.001) associated with lodging resistance, plant height and upper internode diameter (Table 2). Eleven markers (GENE-3066_157, BS00076772_51, RAC875_c103443_475, BS00011514_51, Tdurum_contig97342_ 274, BS00068710_51, Excalibur_c96921_206, Ex_c69054_723, Ra_c6429_1217, BobWhite_c12261_130, Excalibur_c8931_432) sustained the association for several seasons (see Table 2).

**Table 2.**
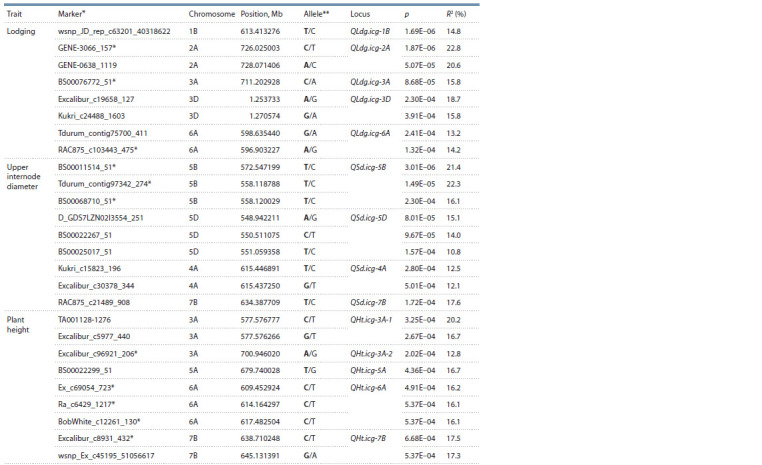
List of the SNP markers associated with lodging resistance, upper internode diameter and plant height in spring soft wheat varieties * Markers are indicated for which associations have been established from the data at least two growing seasons. ** The favorable allele is highlighted in bold.

For the lodging-resistance trait, eight true SNP markers were detected in the five loci located in chromosomes 1В, 2А, 3А, 3D and 6А as per the physical mapping of reference bread wheat variety sequence IWGSC RefSeq v1.0. Highly significant ( p < 0.00002) associations were observed for loci QLdg.icg-1B and QLdg.icg-2A in chromosomes 1B and 2А, respectively (see Table 2). The presence of favorable alleles, on average, increased the lodging resistance by 0.4–0.8 points depending on a locus (Table 3). For the loci in chromosome 3А (711.20 Мb region) and in chromosome 6А (596.90–598.63 Mb), it was found that the increased resistance to lodging of the varieties containing favorable alleles led to their reduction in height by 3 cm. Locus QLdg.icg-3D was mapped in the region of 1.25–1.27 Мb of chromosome 3D, and 8 varieties (Saratovskaya 29, Saratovskaya 42, Lutescens 62, Tulaikovskaya belozernaya, Volgouralskaya, Lutescens 80, Albidum 73, Ilinskaya) carried the unfavorable alleles of markers Excalibur_c19658_127 and Kukri_c24488_1603, whose presence led to statistically significant reduction of the lodging resistance and upper internode diameter (see Table 3)

**Table 3.**
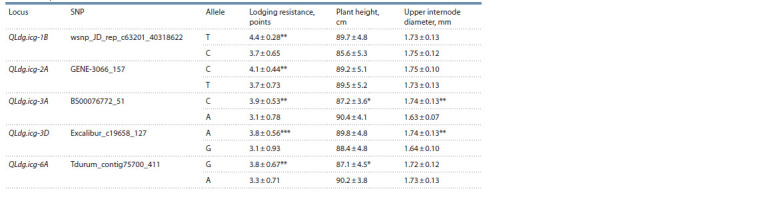
The lodging resistance, upper internode diameter and plant height traits in spring wheat varieties and their dependance on the locus/allele status Notе. Means ± standard deviation (M ± SD) calculated on trait evaluation in 2017–2019. An asterisk indicates the significance of differences between trait parameter for different alleles, * p < 0.05; ** p < 0.01; *** p < 0.001.

Nine SNPs significantly associated with the upper internode diameter were found in the four loci of chromosomes 5B, 5D, 4A and 3A (see Table 2). The GWAS performed demonstrated that the favorable alleles of positive loci were detected in no more than 10 % of studied varieties. For the plant height the most valuable associations were identified in chromosomes 3А, 5А, 6А and 7В (see Table 2). In chromosome 3A, two loci were found, one of which, QHt.icg-3A-1, was localized in the 577.58 Mb region and the other – in the 700.94 Mb region. The QHt.icg-3A-2 locus was of particular interest because it did not only significantly reduce the plant height (by 7.3 cm on average) but also increased the lodging resistance. The favorable alleles of the loci in chromosomes 5А, 6А and 7В were found in the genomes of 11, 90 and 12 % of varieties, respectively and led to plant height reduction by 4 to 5 cm on average.

## Discussion

In the present study, we searched for the genetic factors determining resistance to lodging in a collection of the spring soft wheat varieties selected in Russia. Currently there have been limited number of publications covering the detection of the genetic determinants of this trait due its multigenic character and excessive dependance on environmental factors and plant development stages. Nevertheless, the last two decades have seen the QTLs identified as for lodging resistance as for the stem’s morphological and anatomical parameters that can affect the trait in question.

The classical genetic mapping have enabled one to detect both major and minor loci associated with lodging resistance in the most of the chromosomes of soft wheat such as 1B, 2A, 2D, 3A, 4A, 4B, 4D, 5A, 5B, 6A, 6B, 6D, 7B, 7D (Keller et al., 1999; Hai et al., 2005; Berry P.M., Berry S.T., 2015; Dreccer et al., 2022) as well as the markers located in the vicinity of the target locus. At the same time, it is noteworthy that the detected regions are quite extended due to the limited number of markers that were used while mapping (Börner et al., 2002; Verma et al., 2005).

Genome-wide association mapping has proved to be a more effective method for searching target loci since it requires samples of higher genetic diversity than biparental mapping populations. Moreover, its higher SNP marker coverage enable for more accurate locus mapping and narrower localization regions. In our study, GWAS made it possible to detect five loci in chromosomes 1B, 2A, 3A, 3D and 6A associated with lodging resistance. For the time being, there have been only a few publications, whose authors pursued a similar approach for investigating the genetic factors associated with the trait and with the stem’s anatomical parameters. GWAS has enabled to detect the determinants of lodging resistance in chromosomes 1B, 2A, 3A, 3D, 4B, 5B, 6D and 7А (Cericola et al., 2017; Singh et al., 2019; Akram et al., 2021). According to P.L. Malik et al. (2019) the manifestations of QTLs and their localization in a chromosome also depends on a stage of plant development, so mush so that in early stages (earing), marker– trait associations have been found in chromosomes 1B, 4B, 5B and 7A; and in late stages (maturing) – in chromosomes 1B, 2A, 3D, 4B, 5B and 6D.

Summarizing the published results of genetic and association mapping, a conclusion can be made that the most significant associations for resistance to lodging have been found in chromosomes 3А, 2A and 1B, which matches the data obtained in our study, the only difference being QTL positioning in the chromosomes that depend on the genetic background of the variety material used in the studies. Based on our results, an assumption can be made that the regions of 700–711 and 597–618 Mb of the physical maps of chromosomes 3А and 6А, respectively, can contain clusters of the genes responsible for the plant’s height and their resistance to lodging (see Table 2).

The fact that the loci associated with lodging resistance can have the same localization as those associated with the anatomic parameters has been observed by other authors. According to P.M. Berry and S.T. Berry (2015) the region of 53–82 сМ of the genetic map of chromosome 3A contains a genetic cluster associated with lodging resistance, plant height, internode length/diameter and stem thickness. In other publications a colocalization of the loci associated with lodging resistance and plant height has been noted (Keller et al., 1999; Verma et al., 2005; Malik et al., 2019). Currently, the Catalogue of Gene Symbols for Wheat includes the 25 Rht genes determining plant height (https://shigen.nig.ac.jp/wheat/ komugi/genes/symbolClassList.jsp). Genes Rht-B1, Rht-D1, Rht-8 and their alleles resulting in significant plant height reduction have been mapped in chromosomes 4BS, 4DS, 2DS, respectively (Gale et al., 1975; Korzun et al., 1998; Peng et al., 1999; Chernook et al., 2019). In the present study, the most significant association for the plant-height trait have been found in chromosomes 3А, 5А, 6А and 7В, making it possible to assume that the genomes of the investigated varieties lack highly effective dwarfing genes

The fact that chromosomes 3A, 5А and 7В contain the loci associated with plant height has been confirmed by different authors through genetic mapping and GWAS (Ain et al., 2015; Gao et al., 2015; Akram et al., 2021; Muhammad et al., 2021). Several such genes have been identified in chromosome 6A, some of them (Rht14, Rht16, Rht18, Rht25) found in the short arm, and gene Rht24 – in the long arm (Vikhe et al., 2017; Würschum et al., 2017; Ford et al., 2018; Mo et al., 2018). Genome-wide mapping of the Rht loci in chromosome 6А found genes Rht18 (Ford et al., 2018) and Rht24 (Würschum et al., 2017) in the region of 416–550 Mb of the physical map of the pseudomolecule, which corresponds to the approximate localization of the Qht.icg-6A locus in our study. Unfortunately, there have been no detailed data on the allele composition of dwarfing genes in Russian spring varieties. To verify a correlation between some alleles of the Rht genes and lodging resistance, additional investigations have to be carried out, including those to detect the presence of the Rht genes in the considered variety collection using specific molecular markers.

The found relation between upper internode diameter and lodging resistance is ambiguous. Some authors claim both the length and diameter of both upper and lower internodes in wheat matters for the plant’s resistance to lodging (Berry P.M., Berry S.T., 2015; Packa et al., 2015; Demina, 2019). Others insists this correlation is only valid for the lower internode or absent completely (Zakharov et al., 2014; Zaytseva, Shchennikova, 2020). In the present study, no colocalizations of the loci associated with upper internode diameter and lodging resistance have been detected. It is noteworthy that the correlations between the two traits have been weak, which is probably due to the fact that for the investigated varieties the upper internode diameter plays no significant role for their resistance to lodging.

## Conclusion

Hence, the present study has demonstrated that GWAS is an effective tool for investigating the genetic architecture of a complex trait. Using this method, we have been able to identify several markers associated with lodging resistance, plant height and upper internode diameter in a collection of Russian spring wheat varieties. The obtained results, on the one hand, confirm the conclusions made by other authors about the most critical chromosomes containing the loci responsible for lodging resistance. On the other hand, these results may be important for detecting the samples combining the alleles favorable for several traits for their inclusion into breeding programs.

## Conflict of interest

The authors declare no conflict of interest.
